# Intrahepatic Transposition of Bile Ducts

**DOI:** 10.5402/2012/283527

**Published:** 2012-04-03

**Authors:** Jasmin Delić, Admedina Savković, Eldar Isaković, Sergije Marković, Alma Bajtarevic, Amir Denjalić

**Affiliations:** ^1^Department of Anatomy, Medical Faculty, University Tuzla, 75000 Tuzla, Bosnia and Herzegovina; ^2^Department of Histology and Embriology, Medical Faculty, University Tuzla, 75000 Tuzla, Bosnia and Herzegovina; ^3^Department of Anatomy, Helth Faculty University Zenica, 75000 Tuzla, Bosnia and Herzegovina

## Abstract

*Objective*. To describe the intrahepatic bile duct transposition (anatomical variation occurring in intrahepatic ducts) and to determine the frequency of this variation. *Material and Methods*. The researches were performed randomly on 100 livers of adults, both sexes. Main research methods were anatomical macrodissection. As a criterion for determination of variations in some parts of bile tree, we used the classification of Segmentatio hepatis according to Couinaud (1957) according to Terminologia Anatomica, Thieme Stuugart: Federative Committee on Anatomical Terminology, 1988. *Results*. Intrahepatic transposition of bile ducts was found in two cases (2%), out of total examined cases (100): right-left transposition (right segmental bile duct, originating from the segment VIII, joins the left liver duct-ductus hepaticus sinister) and left-right intrahepatic transposition (left segmental bile duct originating from the segment IV ends in right liver duct-ductus hepaticus dexter). *Conclusion*. Safety and success in liver transplantation to great extent depends on knowledge of anatomy and some common embryological anomalies in bile tree. Variations in bile tree were found in 24–43% of cases, out of which 1–22% are the variations of intrahepatic bile ducts. Therefore, good knowledge on ductal anatomy enables good planning, safe performance of therapeutic and operative procedures, and decreases the risk of intraoperative and postoperative complications.

## 1. Introduction

Biliary drainage has long been called Achilles heel liver transplantion, and biliary complications compromise the succese of liver transplantation [1]. A precise understanding of general anatomic principles and common variations is the key to safe living donor liver transplantation [2]. This procedure requires not only a precise understanding of liver anatomy but also the means of assessing them. One of the most important challenges is that of managing the biliary duct during liver lobe resection and reimplantation. Olos biliary anatomy variants are associated with an increased risk of postoperative complications, including biliary leaks and strictures, in both the donor and recipient [3, 4]. One cause of complications is unrecognized anomalous biliary anatomy (24%–57% of individuals have variant biliary patterns) [5–8]. Although anomalous anatomy is not always a contraindication for liver donation, knowledge of variant anatomy is critical to ensuring the safety of donors and aids selection of suitable candidates [9, 10].

The authors as Varotti et al., Heloury et al., Soares et al., and Cheng et al. noted intrahepatic transposition of bile ducts in 1 to 22% of cases [10–13]. Intrahepatic transposition of bile ducts is manifested by the fact that bile ducts originating from the right liver lobe may end in the left liver duct (ductus hepaticus sinister); that is, bile ducts of the left liver lobe may end in the right liver duct (ductus hepaticus dexter).

## 2. Material and Methods

The researches were performed randomly on 100 livers of adults, both sexes. Main research methods were anatomical macrodissection and statistics. As a criterion for determination of variations in some parts of bile tree, we used the classification Segmentatio hepatis according to Couinaud (1957) according to Terminologia Anatomica, Thieme Stuugart: Federative Committee on Anatomical Terminology, 1988.

## 3. Results

**Figure 1:**
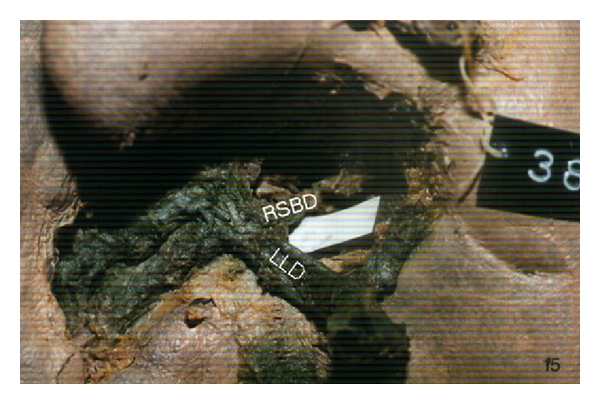
Right-left intrahepatic transposition of bile ducts.

After the researches have been completed, intrahepatic transposition of bile ducts was found in two cases (2%); out of total examined cases (100) right-left transposition and left-right intrahepatic transposition were found. In one case we found right-left intrahepatic transposition, where the right segmental bile duct (RSBD), originating from the segment VIII, joins the left liver duct (LLD). Newly evolved duct is distally united to ductus hepaticus dexter and they form common liver duct (ductus hepaticus communis) (Figure 1).

**Figure 2:**
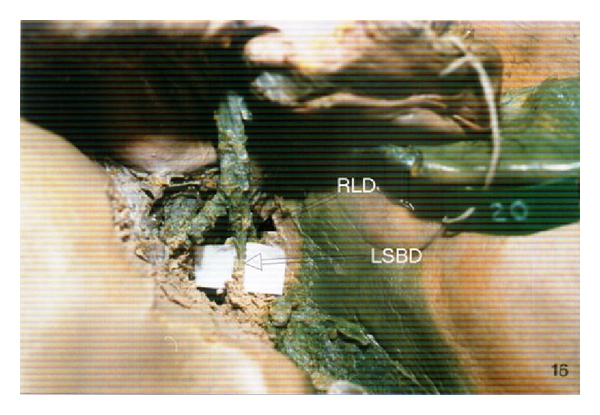
Left-right transposition of intrahepatic bile ducts. Left segmental bile duct enters right liver duct.

In the second case (1%), left-right intrahepatic transposition was observed. Left segmental bile duct (LSBD) originating from the segment IV ends in right liver duct (RLD) (Figure 2).

## 4. Discussion

Variability of form, of position in space, and of topographic relations of bile ducts is immense. Some variations appear very rarely and in small percentages. On the other hand, some of the rare variations of bile ducts are in the focus of surgical anatomy in this area. The key problem in research of variable anatomy of bile ducts is heterogeneity of the sample, as well as the case of a small sample. Because of all the cited, significant disagreements are present concerning the findings of different researchers in the field of bile ducts anatomy, what is contributed by lacking of uniformity of methods and criteria of the research.

Results of this research to a great extent confirm the other authors' results presented in the literature available. The right-left transposition of intrahepatic bile ducts, observed in 1% of cases in our research material, is a more frequent variation and is found, according to the literature available, in 2–22% of cases (bile ducts originating out of segments V, VI, and VII) [14]. The authors as Jin et al., Heloury et al., and Cheng et al. found the left-right transposition in 1–3% of cases, what corresponds to the results of our researches [7, 11].

## 5. Conclusion

Safety and success in liver transplantation to great extent depends on knowledge of anatomy and some common embryological anomalies in bile tree. Variations in bile tree were found in 24–43% of cases, out of which 1–22% are the variations of intrahepatic bile ducts. Therefore, good knowledge on ductal anatomy enables good planning, safe performance of therapeutic and operative procedures, and decreases the risk of intraoperative and postoperative complications.
